# Double‐Antibody Sandwich Enzyme‐Linked Immunosorbent Assay for Detecting Feline Coronavirus Infection

**DOI:** 10.1002/vms3.71289

**Published:** 2026-10-14

**Authors:** Bo Dong, Weijie Zou, Wenqian Hu, Hongxiao Wu, Yaqing Jing, Fuquan Xie, Shuo Zhang, Weiming Lin

**Affiliations:** ^1^ College of Life Sciences of Longyan University Longyan China; ^2^ Engineering Research Center for the Prevention and Control of Animal Original Zoonosis College of Life Sciences Fujian Province University Longyan University Longyan China; ^3^ Fujian Provincial Key Laboratory for the Prevention and Control of Animal Infectious Diseases and Biotechnology Longyan China

**Keywords:** antigen detection, diagnosis, enzyme‐linked immunosorbent assay (ELISA), faecal samples, feline coronavirus

## Abstract

**Objectives:**

Double‐antibody sandwich enzyme‐linked immunosorbent assay (DAS‐ELISA) is a simple and accurate method for detecting viral pathogens. This study established a DAS‐ELISA for the diagnosis of feline coronavirus (FCoV).

**Methods:**

In this study, DAS‐ELISA was performed using FCoV S‐protein rabbit polyclonal and mouse monoclonal antibodies. The optimal reaction conditions were determined using the chessboard method. The specificity, repeatability and receiver operating characteristic (ROC) curve were used to verify the clinical application potential of the DAS‐ELISA.

**Results:**

The intra‐ and inter‐assay %CVs were 3.90–14.10 and 2.55–11.02, respectively. In a preliminary specificity evaluation using 12 faecal samples each from cats infected with feline panleukopenia virus, feline calicivirus or feline herpesvirus, the DAS‐ELISA showed no detectable cross‐reactivity with any of these pathogens. The positive coincidence rate of DAS‐ELISA and RT‐PCR was 81.3%. The ROC curve showed that the sensitivity, specificity and ROC AUC were 81.3%, 80.0% and 0.861, respectively.

**Conclusion:**

Collectively, these results suggest that the developed DAS‐ELISA can be used to detect FCoV pathogens in clinical samples. This study provides a selection method for the diagnosis of FCoV and a data supplement for epidemiological studies of FCoV.

## Graphical abstract

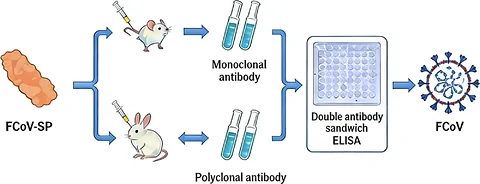

## 1 Introduction

Feline coronavirus (FCoV) is a non‐segmented single‐stranded positive‐strand RNA virus with a genome size of approximately 29 kb (Kipar and Meli 2014). FCoV first appeared in the 1960s, and the disease caused by this virus was first described by Holzworth (1963). However, the viral aetiology was only suggested until 1966 by Wolfe, who performed viral infectivity tests by inoculating healthy animals with tissues from diseased cats (Kennedy 2020). FCoV was confirmed as a member of the genus coronavirus in 1968 (Beesley and Hitchcock 1982; Ward et al. 1968). FCoV causes two distinct disease types and can be distinguished by its biological behaviour rather than morphology. The two‐pathogenic forms have different names, although both are FCoVs (Pratelli 2008). One of these, feline enteric coronavirus (FECV), is highly prevalent in multi‐cat environments and is highly contagious, infecting almost 100% of cats exposed to FECV. However, these infections are mostly asymptomatic or cause only mild transient diarrhoea. In contrast, another form, feline infectious peritonitis virus (FIPV), is not transmitted by the faecal–oral route but is generated by FECV mutations in a small proportion of infected cats, leading to lethal feline infectious peritonitis (FIP). The replication site of FIPV is no longer limited to the gut; it can replicate in macrophages and cause diseases. After infecting cat peritoneal macrophages, FIPV continues to infect other susceptible cells in the body and produces progeny viruses with higher titres (Stoddart and Scott 1989). It has been reported that mutations in the FCoV genome play an important role in the development of FIP. These mutations are responsible for the changes in viral tropism from intestinal cells to monocytes/macrophages (Felten and Hartmann 2019). However, the genes that produce the mutations that lead to FIPV development still need to be explored more. As FECV has the risk of mutating into FIPV, accurate diagnosis of FCoV is crucial for antiviral treatment.

The spike protein (S) of FCoV is a Type I transmembrane protein, which is divided into the N‐terminal (S1) domain responsible for receptor binding and the C‐terminal (S2) domain containing fusion peptide. The S protein plays an important role in the pathogenesis of FIP, is a key determinant of phagocytosis, and is also the main antigen that the virus induces the body to produce neutralizing antibodies. The S protein contains a recognized fusion peptide, which is mediated into the host cell through the S protein, and FCoV envelope fuses with the host cell membrane (Jaimes and Whittaker 2018; Bosch et al. 2003). The S protein is the main basis for antigen classification. FCoVs can be divided into Type I and II serotypes according to their S proteins (Lewis et al. 2015). Of the two serotypes, Type I FCoV strains have high prevalence in both FCoV‐infected cats and clinically healthy cats. The S protein contains abundant epitopes that mediate the production of antibodies by the infected host and plays an important role in antigen recognition (Sun et al. 2008). Therefore, the early detection of coronaviruses largely relies on the S protein, and several studies have reported a positive effect of the S protein on the detection of some coronaviruses (Gui et al. 2018; Márquez‐Ipiña et al. 2021; Peng et al. 2022). However, few FCoV detection methods based on S‐protein‐specific monoclonal antibodies (mAbs) have been reported.

Several FCoV detection methods have been reported (Guan et al. 2020; Pratelli et al. 1999; Xiao et al. 2022). Real‐time quantitative polymerase chain reaction (RT‐qPCR) using viral RNA‐specific primers, high sensitivity and simple experiments is a better diagnostic method. However, false‐negative results due to novel variants and false‐positive results due to sample contamination still exist (Nicolini et al. 2017). In addition, RT‐qPCR can only be performed in fully equipped laboratories. Other serological methods can detect FCoV immunoglobulins (IgG, antibodies (Abs)) (Dong et al. 2021). Because IgG antibodies are produced several days after viral infection, this is different for each patient (O Murchu et al. 2021). Therefore, establishing a method for the early diagnosis of FCoV infection is necessary.

In this study, we developed a dual‐antibody sandwich enzyme‐linked immunosorbent assay (DAS‐ELISA) as a sensitive, specific and easy‐to‐perform viral antigen assay for high‐throughput detection. Using a previously obtained truncated Type I FCoV S protein as the immunogen (Dong et al. 2021), we generated monoclonal and polyclonal antibodies to establish a DAS‐ELISA capable of detecting the Type I FCoV S protein in faecal samples and ascitic fluid. DAS‐ELISA has acceptable reliability, specificity, repeatability and sensitivity and is suitable for FCoV detection.

## 2 Methods

### 2.1 Preparation of FCoV‐SP Protein and Immunization of Animals

The FCoV S‐protein fragment, FCoV‐SP, was expressed and purified. The purified protein was used as an immunogen to immunize mice and rabbits, as previously reported (Dong et al. 2021). Briefly, the BALB/c mice (6‐week‐old) procured from Wu's Experimental Animal Trading Co. Ltd. (Fujian, China) were housed under standard and ventilated conditions in the animal care facility of Longyan University. The Freund's complete adjuvant (CFA) + FCoV‐SP was prepared by slowly adding CFA to the FCoV‐SP antigen at a final ratio of 1:1 (v/v) followed by gentle shaking. These mice were subcutaneously injected with purified CFA + FCoV‐SP with 50 µg/mouse by weekly injections for 3 weeks. Three adult (12‐week‐old) rabbits were also immunized with 200 µg/rabbit of FCoV‐SP according to the mice immunization protocol. Blood samples were collected from the tail veins of mice or the auricular arteries of rabbits. Antibody titres were determined using indirect ELISA (iELISA).

After the last injection, whole blood was collected from the auricular vein of the rabbit with the highest antibody titre, and serum was obtained. Immunoglobulin G was purified from the antiserum with a protein A Sepharose column using a Pure‐1A antibody purification kit, according to the manufacturer's instructions (Sigma, USA). The purified polyclonal antibody was stored at −80°C.

### 2.2 Identification of mAbs Against FCoV‐SP Protein

After the last immunization, antibody titres in the serum were detected using ELISA. Myeloma cells and immune mouse spleen cells were fused by conventional methods with a cell number ratio of 1:8, and 50% PEG3350 was used as the fusion agent. The cells were inoculated into 96‐well culture plates containing feeder cells; each well was 100 µL and cultured in a 5% CO_2_ saturated humidity temperature chamber. At Days 3 and 6 after fusion, HAT culture medium was used for fluid change; HT culture medium was used for fluid change on Days 9 and 12, and normal complete culture medium was used after Day 15. An iELISA method was established to determine the optimal amount of antigen coated by checkerboard method, and the coated antigen was the purified recombinant protein. iELISA method was used to detect the supernatant of hybridoma cell culture holes, and the positive hybridoma cells were cloned by limited dilution method until the screening result was 100% positive. Male BALB/c mice aged 8–12 weeks were injected intraperitoneally with 0.5 mL/FICA. Seven days later, 1 × 10^5^ hybridoma cells were injected into the abdomen to observe the status of the mice. After 8–15 days of tumour growth, ascites was collected, and the cells obtained by ascites centrifugation were injected into another mouse injected with paraffin to continue to produce ascites antibodies. The titre was measured by iELISA and stored at −70°C. The isotypes of the obtained mAbs were determined using a commercial mouse mAb isotyping kit (Zymed Laboratories, Carlsbad, CA, USA).

### 2.3 Rabbit Polyclonal Antibody Preparation

Two weeks after the last injection, whole blood was collected from the auricular vein of the rabbit with the highest antibody titre, and serum was obtained. Immunoglobulin G was purified from the antiserum with a protein A Sepharose column using a Pure‐1A antibody purification kit, according to the manufacturer's instructions (Sigma, USA). The purified polyclonal antibody was stored at −80°C.

### 2.4 Establishment and Optimization of DAS‐ELISA

A checkerboard titration was used to determine the best working concentrations for the capture and detection antibodies. The purified polyclonal antibody was serially diluted twofold from 0.60 to 0.02 µg/mL in a coating solution. The purified FCoV SP was serially diluted twofold from 3.25 to 0.20 µg/mL, and the mAb was serially diluted twofold from 2.0 to 0.03 µg/mL. The coating time, sealing conditions, antigen and antibody incubation times and colour development time were tested to determine the optimal reaction condition. To determine the analytical limit of detection (LOD) of the DAS‐ELISA, purified FCoV‐SP antigen was serially diluted twofold from 3.25 to 0.0127 µg/mL in PBS, and each dilution was tested in triplicate using the optimized assay conditions. A standard curve was generated by plotting the mean OD_450_ values against the logarithm of the antigen concentration. For checkerboard titrations, two controls were used: PBS blank (baseline) and RT‐PCR‐negative faecal samples (matrix control). Optimal antibody concentrations were selected by the highest P/N ratio, defined as (OD_450_ positive − OD_450_ blank)/(OD_450_ negative − OD_450_ blank), with minimal background. Matrix validation used FCoV‐SP spiked into negative faecal matrix at three concentrations (3.25, 0.80 and 0.20 µg/mL); recovery rates were calculated against matrix blank, and inter‐dose CVs were assessed. This ensured robustness against background and matrix interference, improving reproducibility.

Briefly, microplates were coated with each capture polyclonal antibody at a concentration of 0.15 µg/mL (50 µL/well) overnight at 4°C. After blocking, antigen samples, positive controls (FCoV‐SP) or negative controls identified using PCR were distributed into the wells in duplicate (50 µL/well), and the plates were placed at 25°C ± 5°C for 45 min. After washing, the mAb was added at a concentration of 1.0 µg/mL (50 µL/well), and the plates were incubated at RT for 30 min. After three washes, the HRP‐conjugated goat anti‐mouse IgG was diluted to 1:10,000. Fifty microlitres of the diluted antibody were added to each well for ELISA, followed by incubation at RT for 60 min. Following incubation and washing, the reaction was developed with 50 µL of 3,3,5,5‐tetramethylbenzidine (TMB) solution (Invitrogen, USA). The reaction was stopped with 50 µL of 2 M H_2_SO_4_, and the absorbance was recorded at 450 nm.

### 2.5 Determination of the Cut‐Off Value for the DAS‐ELISA

Twenty FCoV‐negative faecal samples were obtained from healthy cats that were also diagnosed without FCoV infection using RT‐PCR. These faecal samples were placed in a 2 mL centrifuge tube, diluted with PBS, swirled at room temperature at 3000 × *g* for 5 min and centrifuged at room temperature at 1200 × *g* for 10 min to extract the supernatant. Then, supernatant was treated with 1% Triton X‐100 and 0.3% tri‐*n*‐butyl phosphate (TNBP) for 2 h at RT to inactivate the virus and was then detected using the established DAS‐ELISA. The cut‐off value for DAS‐ELISA was calculated using the mean OD value (*X*) of 20 FCoV‐negative samples detected using RT‐PCR plus three standard deviations (SDs). When the OD_450_ of the sample to be tested was greater than or equal to the cut‐off value, it was determined to be positive. In contrast, the results were negative.

### 2.6 Check for the Coefficient of Variation

To assess the DAS‐ELISA developed in this study, the intra‐ and inter‐assay variations were determined to confirm reproducibility. To investigate the precision of the evaluation, five samples (three positive and two negative) were analysed in duplicate within the same assay and repeated over a 3‐day period. The average, SD, median and CV were calculated using the Statistics Package for Social Science 19.0 (SPSS 19.0).

### 2.7 Specificity of the DAS‐ELISA

To evaluate the specificity of the DAS‐ELISA, faecal samples from cats infected with feline panleukopenia virus (FPV), feline calicivirus (FCV) or feline herpesvirus (FHV) were tested using DAS‐ELISA, and the specificity results were determined according to the cut‐off value.

### 2.8 Detection of Faecal Samples Using the DAS‐ELISA and RT‐PCR

Sixty‐two faecal samples were collected from cats in Longyan, Xiamen and Fuzhou, Fujian, China. Informed consent was obtained from the cat owners prior to sample collection, and the cat owners approved the sampling and data release. The samples were tested using DAS‐ELISA and RT‐PCR to determine the agreement rate of the CDV detection results obtained from clinical samples. The PCR primers were, respectively, forward primer (5′‐TGCTTCGGCTAACTTTGGTG‐3′) and reverse primer (5′‐CAATCATCTCAACCTGTGTGTCAT‐3′) as previously described (Guan et al. 2020). The specific procedure was as follows: 3 min at 95°C, followed by 35 cycles of 15 s at 95°C, 1 min at 56°C and 30 s at 72°C. The agreement between the RT‐PCR and DAS‐ELISA techniques was measured using the kappa statistic (Tang et al. 2015).

### 2.9 Western Blotting

The purified protein was transferred to polyvinylidene fluoride membranes in the transfer buffer and resolved by sodium dodecyl‐sulphate polyacrylamide gel electrophoresis. Nonspecific binding was blocked with Tris‐buffered saline containing 5% Tween 20 and 5% skim milk at room temperature for 1 h. The membranes were incubated overnight at 4°C with the internal control 1:500‐diluted monoclonal antibody or 1:500‐diluted polyclonal antibody to FCoV‐SP. They were subsequently incubated with dilutions of anti‐mouse or anti‐rabbit HRP‐conjugated secondary antibody (ab6728; Abcam, Cambridge, UK or ab6721; Abcam, Cambridge, UK). The recombinant protein was detected, and a predicted molecular weight of 32 kDa was confirmed by western blotting.

### 2.10 Statistical Analysis

The average, SDs, medians and CVs were calculated using the software Statistics Package for SPSS 19.0 software (version 19.0; SPSS Inc., Chicago, IL, USA). The diagnostic sensitivity and specificity were evaluated by receiver operator characteristic (ROC) analysis using SPSS 19.0 software. All data were analysed and visualized in using GraphPad Prism 8.0 (GraphPad Software, CA, USA).

## 3 Results

### 3.1 Western Blot Analysis Using mAbs and Polyclonal Antibodies

Antibody‐positive subclones were obtained using the limited dilution method (Wang et al. 2021). Ten days after the test, positive clones were isolated continuously, and subcloning was performed using the limited dilution method. A positive subclone, E5, was identified and used to produce mAbs against ascites. The E5 monoclonal isotype was identified as IgG2b. FCoV‐SP was identified using western blotting. The results showed that E5 mAb could recognize FCoV‐SP, which has a relative molecular weight of approximately 32 kDa (Figure 1). Therefore, the prepared mAbs could specifically recognize FCoV‐SP proteins. Additionally, the reactivity of rabbit polyclonal antibody against FCoV‐SP was identified by western blotting. As shown in Figure 2, the rabbit polyclonal antibody against FCoV‐SP could specifically react (Figure 2).

**FIGURE 1:**
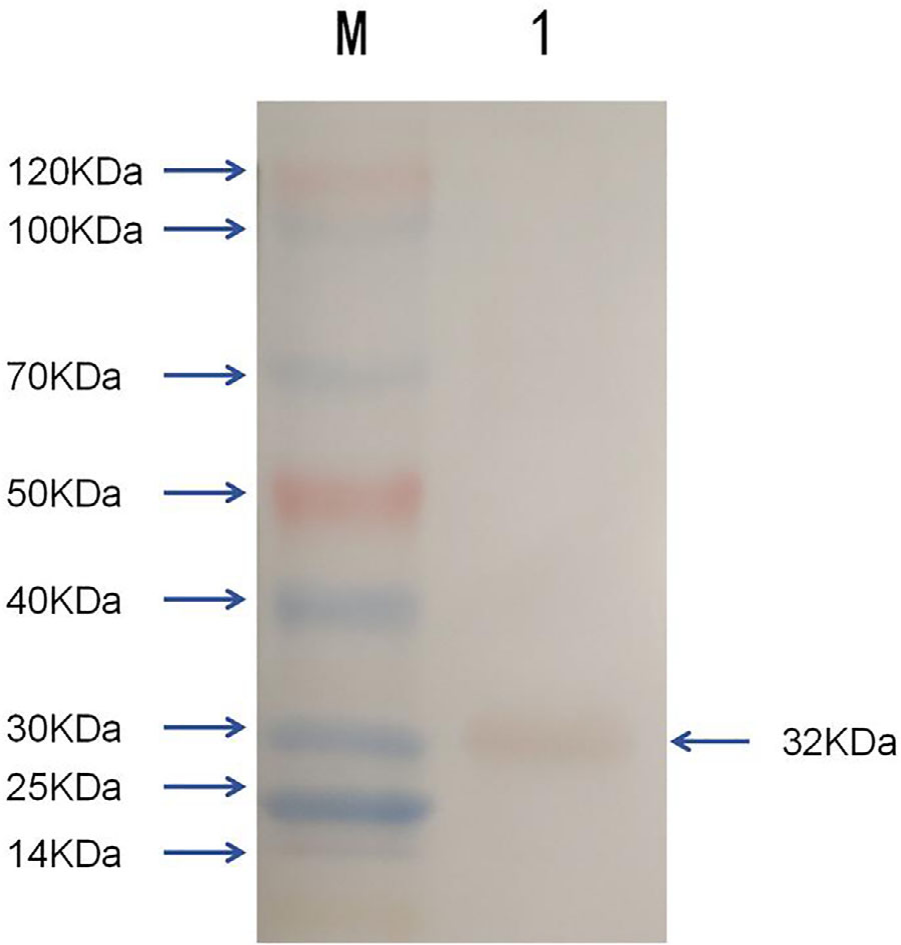
Western blot analysis of the monoclonal antibodies (mAbs) against feline coronavirus spike protein (FCoV‐SP). Lane M: protein marker (14–120 kDa); Lane 1: the purified FCoV‐SP.

**FIGURE 2:**
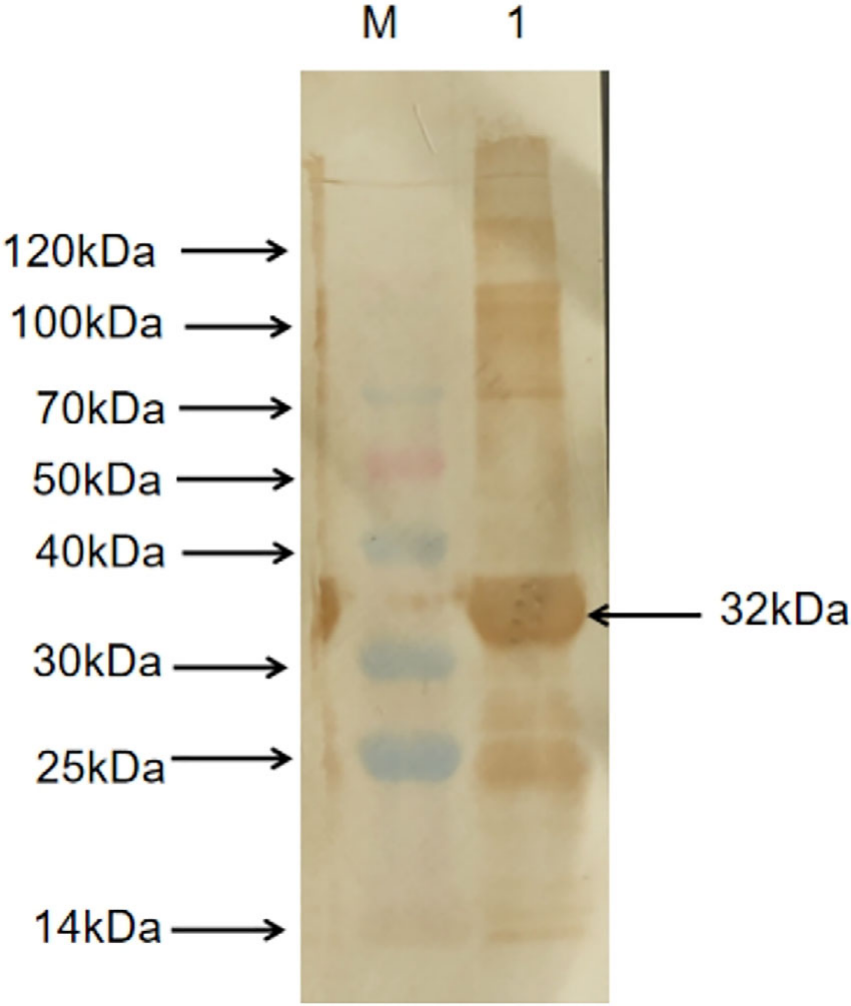
The reactivity of the polyclonal antibody with FCoV‐SP was identified by western blotting. Lane M: protein marker (14–120 kDa); Lane 1: the purified FCoV‐SP.

### 3.2 Determination of the Cut‐Off Value

Twenty negative faecal samples were tested to determine the cut‐off value for the iELISA. The average OD_450_ value of negative samples was 0.33, the SD is 0.17, and the cut‐off formula is *X* + 3 × SD. Therefore, the cut‐off value is 0.85. When the OD_450_ value of the sample to be tested was higher than 0.85, it was determined as positive, whereas those less than or equal to 0.85 were considered negative (Figure 3). Using the standard curve generated from serially diluted purified FCoV‐SP antigen (*R* ^2^ = 0.991), the analytical LOD of the DAS‐ELISA was determined to be approximately 0.15 µg/mL, calculated as the antigen concentration corresponding to the mean OD_450_ of 20 negative faecal samples plus 3 SDs (*X* + 3 × SD).

**FIGURE 3:**
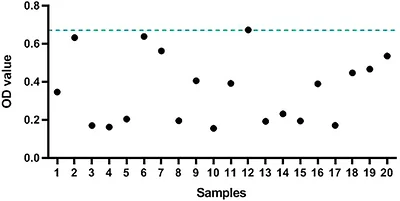
Determination of the cut‐off value of the double‐antibody sandwich enzyme‐linked immunosorbent assay (DAS‐ELISA). *n* = 3. The blue dashed line indicates the cut‐off value (0.85).

### 3.3 Repeatability of DAS‐ELISA

The results of the repeatability tests are presented in Table 1. The intra‐ and inter‐assay %CVs were 3.90–14.10 and 2.55–11.09, respectively. Notably, Sample 1 showed an intra‐assay %CV of 14.07, which marginally exceeded the conventional 10% acceptance criterion commonly recommended for ligand‐binding assays in regulated bioanalysis. This elevated variation may be attributed to the relatively high OD_450_ value of this sample, where small inconsistencies in pipetting or washing steps could be proportionally amplified. Nevertheless, the remaining four samples demonstrated intra‐assay %CVs below 8.66, and the inter‐assay %CVs for all samples were below 11.09. In general, all %CV values were <20, indicating acceptable repeatability of DAS‐ELISA for preliminary validation purposes (DeSilva et al. 2003).

**TABLE 1:** Inter‐ and intra‐reproducibility of double‐antibody sandwich enzyme‐linked immunosorbent assay (DAS‐ELISA).

	Sample no.	Times	Average	Standard deviation	%CV
Intra‐assay	1	3	1.17	0.17	14.07
	2	3	1.12	0.10	8.66
	3	3	1.07	0.05	5.01
	4	3	0.08	0.00	3.90
	5	3	0.05	0.00	5.76
Inter‐assay	1	3	1.18	0.10	8.26
	2	3	1.11	0.09	8.07
	3	3	1.07	0.12	11.09
	4	3	0.08	0.01	6.13
	5	3	0.04	0.00	2.55

### 3.4 Specificity of DAS‐ELISA

The specificity of the DAS‐ELISA was assessed by testing faecal samples from three other viruses: FPV, FCV and FHV. A total of 48 faecal samples were tested. Among them, 12 samples were FCoV‐infected cats, 12 samples were FPV‐infected cats, 12 samples were FCV‐infected cats, and 12 samples were FHV‐infected cats. The results showed that only FCoV‐infected samples and FCoV‐SP proteins were positive, and no positive results or cross‐reactions were detected for other viruses (Figure 4). The results showed that the DAS‐ELISA was specific for FCoV.

**FIGURE 4:**
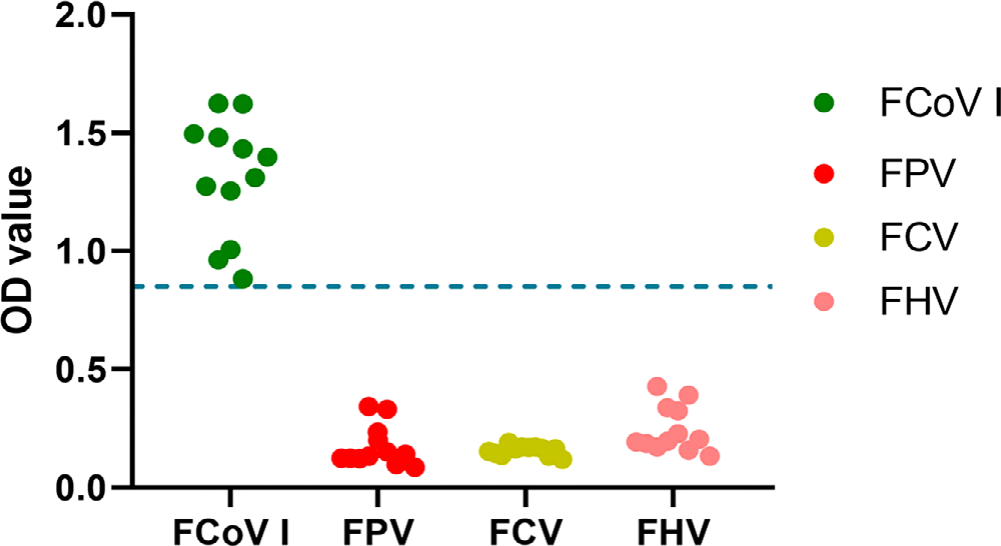
Specificity test of the double‐antibody sandwich enzyme‐linked immunosorbent assay (DAS‐ELISA). The blue dashed line indicates the cut‐off value (0.85). FCoV, feline coronavirus; FCV, feline calicivirus; FHV, feline herpesvirus; FPV, feline panleukopenia virus.

### 3.5 Clinical Sensitivity and Specificity of DAS‐ELISA

DAS‐ELISA was used to detect FCoV in 62 faecal samples that had been detected using RT‐qPCR. Of the 32 positive samples, all but 6 (26/32 = 81.3%) were correctly identified relative to the DAS‐ELISA. Of the 30 negative samples, 25 samples (25/30 = 83.3%) were correctly identified (Table 2). The ROC curve showed a sensitivity of 81.3%, a specificity of 80.0% and an area under the ROC curve (ROC AUC) of 0.861 (Figure 5).

**TABLE 2:** Results for faecal samples that were tested to compare between RT‐PCR and double‐antibody sandwich enzyme‐linked immunosorbent assay (DAS‐ELISA).

	RT‐PCR		
DAS‐ELISA	Positive	Negative	Total
Positive	26	5	31
Negative	6	25	31
Total	32	30	62

**FIGURE 5:**
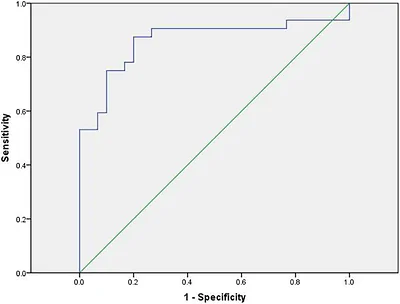
The receiver operating characteristic (ROC) curve generated using RT‐PCR as the diagnostic standard.

## 4 Discussion

FCoV is a well‐known coronavirus in veterinary medicine and has received considerable attention from researchers (Paltrinieri et al. 2021). FCoV Type I is the most primitive FCoV and the most prevalent serotype worldwide (Malbon et al. 2019). On the basis of current clinical manifestations, FCoV is difficult to detect. Nevertheless, early diagnosis of FCoV infection is important as it is an important measure to treat and control future outbreaks. Although virus isolation and identification is currently the most commonly used pathogen detection method, due to the difficulty of FCoV in vitro culture, the long isolation and culture time, and the high requirements for the experimental operation environment. Therefore, it is not suitable for large‐scale detection requirements. Furthermore, although RT‐PCR meets the requirements of sensitivity and rapidity in antigen detection, precisely because of its excessively high sensitivity, false positive results may occur, and it has high requirements for the experimental operating environment, making it difficult to be promoted in large‐scale clinical diagnosis. ELISA, as a low‐cost, sensitive and fast diagnostic method, has become an acceptable method in the study of disease aetiology and serological diagnosis.

Currently, FCoV ELISA detection mainly uses iELISA to study the titre of FCoV antibodies in cat serum to determine the infection situation. However, the use of ELISA for the diagnosis of FCoV has rarely been reported. DAS‐ELISA can accurately quantify antigens at the protein level, and the experimental operation is simple (Ten Haaf et al. 2017), providing an accurate and sensitive method for the detection of viral pathogens that can be further applied to the detection of FCoV. Therefore, we developed a DAS‐ELISA to detect pathogens and validated its clinical application potential. One influencing factor of the double antibody sandwich ELISA detection method is the specific binding of the pathogen to the antibody. In this study, polyclonal antibodies against rabbit FCoV S protein were used as capture antibodies. This polyclonal antibody has multiple binding sites of different epitopes and can bind to multiple antigenic epitopes on the surface of the antigen to be tested, thereby increasing the binding probability with the antigen. The monoclonal antibody against S protein in mice was used as the detection antibody, ensuring the sensitivity and specificity of the ELISA established in this study.

Specificity experiments showed that the established DAS‐ELISA could not detect other feline viruses but had good specificity. In addition, the results showed that the maximum value of intraplate CV was 14.07% and the maximum value of interplate CV was 11.09%, both less than 20%, indicating that ELISA had good repeatability. In addition, the reproducibility assessment revealed that most samples yielded intra‐ and inter‐assay %CVs below 10, with the exception of one positive sample showing an intra‐assay %CV of 14.07. Although this single elevated value marginally exceeds the ideal 10% threshold commonly recommended for ligand‐binding assay validation, it remains below the 20% upper limit generally accepted for exploratory immunoassays (DeSilva et al. 2003). The observed variability in high‐signal samples may reflect inherent technical fluctuations at elevated absorbance ranges, where small inconsistencies in pipetting or washing steps could be proportionally amplified. Nevertheless, the remaining four samples demonstrated intra‐assay %CVs below 8.66, and the inter‐assay %CVs for all samples were below 11.09, collectively indicating acceptable repeatability of DAS‐ELISA for preliminary validation purposes.

To assess the clinical potential of the DAS‐ELISA, we compared the RT‐PCR results with the DAS‐ELISA results using 62 cat faecal samples. The results showed that the positive coincidence rate was 81.3%, the clinical sensitivity was 78.1%, the clinical specificity was 80.0%, and the AUC was 0.861, indicating that this method could be applied to the pathogen diagnosis of FCoV. However, 26 of the 62 samples had inconsistent test results, 6 of which tested positive by RT‐PCR and negative by ELISA. Studies have pointed out that samples with weak RT‐PCR‐positive results are usually identified as positive, whereas in ELISA, as long as the OD value is lower than the cut‐off value, the sample is considered negative, which may also be one of the reasons for the error in the two test results (Felten et al. 2020). In the early stages of FCoV infection, the content of FCoV in the bodies of infected cats is low, and the antigen content in the faeces is low. Therefore, it is difficult to detect FCoV using ELISA in the early stages of infection and recovery, so there may be negative ELISA detection and positive RT‐PCR detection. Additionally, five samples were negative tested by RT‐PCR but positive tested by ELISA. Notably, the OD_450_ values of these five false‐positive samples were clustered just above the cut‐off, rather than being strongly positive, suggesting low‐level matrix interference rather than specific antigen‐antibody binding. This might be because these faecal samples contain endogenous horseradish peroxidase contaminants or other non‐specific matrix components, causing the test results to be non‐specific positive. Although our Triton X‐100/TNBP pre‐treatment partially reduces matrix effects, residual false positives remain. Future optimization should include a peroxidase‐blocking step or inhibitors in the sample diluent. For clinical use, equivocal samples should be confirmed by RT‐PCR.

The present study had several limitations. Because there is currently no FCoV antigen detection ELISA in the market, this study lacks the same or similar commercial ELISA kits for comparative validation. Therefore, this study could only compare the published PCR results with the DAS‐ELISA results and analyse their relative specificity and sensitivity through ROC curve analysis. Additionally, the specificity panel, 12 samples per virus (FPV, FCV and FHV), remains too small to calculate robust 95% confidence intervals for specificity or to definitively rule out clinical cross‐reactivity. A sample size of 30 or more per pathogen is generally recommended for reliable specificity estimation. Therefore, the current specificity data should be interpreted as preliminary, and future validation studies with larger cohorts are necessary to confirm the lack of cross‐reactivity. Moreover, the comparative analysis with other antigen detection techniques, such as isothermal amplification and CRISPR‐based diagnostics, is necessary in order to further determine the diagnostic application prospects of DAS‐ELISA. In addition, although sequence analysis indicated low homology between our Type I‐derived FCoV‐SP and Type II FCoV/CCV, we did not systematically validate the assay against Type II clinical isolates or CCV samples, and such cross‐reactivity testing is warranted in future studies. Furthermore, only cross‐reactivity with infectious viruses commonly found in cat populations was examined in the specific experiment, and more viruses need to be detected in future studies, such as SARS‐CoV‐2, which is transmitted through cat faeces. Furthermore, the intra‐assay %CV of one sample was 14.07, higher than 10. Therefore, more samples need to be selected in future studies to verify the repeatability of DAS‐ELISA. Finally, the analytical LOD (0.15 µg/mL) was determined using purified antigen in buffer, not spiked into faecal matrix. The true clinical LOD may be higher due to matrix effects, and future studies should determine matrix‐adjusted LOD using spiked faecal samples. Although this LOD may be higher than some commercial kits targeting other pathogens, our clinical testing (32/62 positives) confirms its sufficient sensitivity for detecting FCoV during active infection.

In this study, a mouse monoclonal and rabbit polyclonal antibodies were used as capture and detection antibodies, respectively, to develop a DAS‐ELISA that can be used for viral antigen detection. This method does not cross‐react with feline viral pathogens such as FPV, FCV or FHV. In addition, the results of cat faecal samples showed an acceptable coincidence rate between DAS‐ELISA and RT‐qPCR, indicating acceptable sensitivity, specificity and potential as a diagnostic tool for FCoV infection. However, DAS‐ELISA still needs to be tested on more samples to confirm its clinical application.

## Author Contributions

Conceptualization: Bo Dong. Funding acquisition: Bo Dong and Weiming Lin. Methodology: Wenqian Hu, Weijie Zou, Hongxiao Wu, Yaqing Jing, Fuquan Xie and Shuo Zhang. Software: Wenqian Hu and Weijie Zou. Supervision: Bo Dong. Validation: Weiming Lin. Writing – original draft: Bo Dong, Wenqian Hu and Weijie Zou. Writing – review and editing: Bo Dong and Weiming Lin.

## Funding

This study was supported by Fujian Natural Science Foundation General Project (2023J01991), Fujian University Students Innovation and Entrepreneurship Training Program (s202411312019 and s202611312018), Longyan City Science and Technology Plan Project (2023LYF17114) and the Research Fundation of Engineering Research Center for the Prevention and Control of Amal Original Zoonosis, Fujian Province University (Grant No. 2023K02).

## Ethics Statement

All experimental animals were performed after obtaining ethics approval from the Committee on the Ethics of Animal Experiments of Longyan University (LY2023001L). This study was conducted in compliance with the ARRIVE, international (OIE Terrestrial Animal Health Code) and national (CNAS‐CL06:2018) guidelines.

## Conflicts of Interest

The authors declare no conflicts of interest.

## Data Availability

The data that support the findings of this study are available from the corresponding author upon reasonable request.
